# Conservation of pattern as a tool for inference on spatial snapshots in ecological data

**DOI:** 10.1038/s41598-017-17346-6

**Published:** 2018-01-09

**Authors:** Michael A. Irvine, James C. Bull, Matt J. Keeling

**Affiliations:** 1 Institute of Applied Mathematics, University of British Columbia, Vancouver, V6T 1Z2 Canada; 2 Department of Biosciences, Wallace Building, Swansea University, Swansea, SA2 8PP UK; 3 Zeeman Institute (SBIDER), Maths Institute & School of Life Sciences, University of Warwick, Coventry, CV47AL UK

## Abstract

As climate change and other anthropogenic factors increase the uncertainty of vegetation ecosystem persistence, the ability to rapidly assess their dynamics is paramount. Vegetation and sessile communities form a variety of striking regular spatial patterns such as stripes, spots and labyrinths, that have been used as indicators of ecosystem current state, through qualitative analysis of simple models. Here we describe a new method for rigorous quantitative estimation of biological parameters from a single spatial snapshot. We formulate a synthetic likelihood through consideration of the expected change in the correlation structure of the spatial pattern. This then allows Bayesian inference to be performed on the model parameters, which includes providing parameter uncertainty. The method was validated against simulated data and then applied to real data in the form of aerial photographs of seagrass banding. The inferred parameters were found to be able to reproduce similar patterns to those observed and able to detect strength of spatial competition, competition-induced mortality and the local range of reproduction. This technique points to a way of performing rapid inference of spatial competition and ecological stability from a single spatial snapshots of sessile communities.

## Introduction

The rapid assessment of the underlying dynamics of ecological environments is becoming an increasingly important area of research as climate change and other anthropogenic influences increase the uncertainty of ecosystem dynamics^1,2^. Single spatial snapshots taken from remote sensing data such as satellite imagery and aerial photography provide high resolution estimates of vegetation density as well as their spatial correlation structure and interaction with other vegetative communities^3^. It would therefore be highly informative to be able to infer the dynamics of the spatial process based on a single snapshot alone without the need for long-term study of vegetation dynamics^4^.

Techniques for analysis of spatial pattern in ecology have largely focused on either their statistical properties^5,6^ or how their patterns relate to deterministic models of pattern formation^7,8^ (See^9^ for a review of pattern formation in plant-based ecosystems). Techniques have been explored to identify anisotropy and correlations as well as regular pattern phenomena. Models of vegetative pattern formation have been used to compare to patterns found in nature and model properties have been explored, in particular their bifurcation points, in order to discern an ecosystem’s extinction risk, or risk of transition to a barren state^10–12^. Methods such as estimation of spatial variance and skewness have also been used as heuristics for changes in the environmental dynamics and have shown to be theoretically capable of being used as an early warning indicator of a dynamic phase transition^11^. However, these techniques rely on several snapshots through time being available to detect change. Finally, other techniques use Markov Chain Monte Carlo (MCMC) methods that rely on two or more spatial snapshots in time to infer dynamics between them^13,14^. Our approach is to use the properties of the spatial pattern to fit a stochastic vegetation model with spatial competition to a single snapshot of a spatial pattern based on Keeling *et al*.^15^.

A spatial ecological model will typically have many degrees of freedom. For a lattice based system of occupied or unoccupied patches, the number of ways a system can be in is typically large, scaling as 2^*n*×*n*^ for a square lattice of length *n*. As a system begins to evolve however, the degrees of freedom are rapidly reduced until the dynamics are dependent upon a few slow-moving state variables^16^. The challenge is to then find meaningful slow-moving variables that capture the general dynamics. For a vegetation ecosystem, spatial correlation functions are one such set of observables^17^. These will typically evolve at a rate much slower than the more transient observables of the system. Since the underlying dynamics of the system are stochastic, we can then construct the expected rate of change (*ξ*) of the observable *ϕ* of the current system state *S*_*t*_ by considering all the ways in which the system can leave this state,

1 $$\xi :=\frac{\partial }{\partial t}{\mathbb{E}}[\varphi ({S}_{t})]=\sum _{S^{\prime} }{\rm{\Delta }}\varphi ({S}_{t}\to S^{\prime} ){\rm{Rate}}({S}_{t}\to S^{\prime} )\mathrm{.}$$

The expected rate of change of an observable is therefore the sum over all lattice sites *i* of the rate at which an event occurs multiplied by the change in the observable conditioned on the event occurring. This equation may be derived from the Kolmogorov-forward equation (see Supplementary information). An evolving system may have many such observables that are of interest. If such a collection ${\rm{\Phi }}=\{{\varphi }_{1},\ldots ,{\varphi }_{k}\}$ exists then the expected rate of change can be defined for each and the collection of them can be studied. This can be achieved by assuming statistical stationarity, where the rate of change (*ξ*) is zero and subsequently all terms in the right-hand side cancel.

The primary method outlined here is to construct a synthetic (pseudo) likelihood based upon spatial summary statistics^18^. The expected change depends upon the rates at which events occur in the lattice and hence is dependent upon the parameters of the model. If a given system is at statistical equilibrium, all of its salient observables, such as spatial correlations, will be approximately constant in time. This corresponds to when the expected change in the observables are zero. We can thus calculate a total deviation in the rates from the statistically stationary state for a given set of model parameters. The associated likelihood can then be defined as some probability density function that takes the total deviation from statistical stationarity as its value. Once we have such a likelihood, the system is then amenable to model-fitting methods such as likelihood maximization as well as MCMC and other Bayesian techniques. In order to elucidate these ideas we study the case of banded regular pattern formation in ecology and construct a probabilistic cellular automata lattice model that can be used in this approach.

We approach the model fitting in a Bayesian scheme. Bayesian inference can regularize fitting through the use of prior information either from distinct aspects of the data or other data sources. This allows fitting to be performed where the underlying inference problem may be ill-posed due to conflated parameters, lack of data or a likelihood that is uninformative to one or more parameters. The framework also allows sampling to be performed where the space of well-fitting parameters can be investigated, particularly if the likelihood landscape is complex (e.g. due to multi-modality). Disadvantages can come from when priors are misspecified or if the likelihood is computationally intensive as typically many samples are required^19,20^.

In this paper we outline a technique for performing model inference on a spatial snapshot and explore a vegetation-based ecosystem using this technique. We develop and explore a stochastic cellular automata model^21,22^, with local and long-range interaction that produces banding patterns in agreement with a number of ecosystems that exhibit long-range competition. We introduce a technique of fitting a model to a spatial snapshot using a synthetic likelihood-based MCMC approach. We then explore real data in the form of an aerial photographic survey of seagrass meadows in the Isles of Scilly, UK that display banding phenomena applicable to our model.

## Methods

### Model Development

Regular pattern formation is abundant in vegetative communities: including patches, labyrinths, bands and gaps^23^. The leading explanation of this type of pattern formation is due to some separation of spatial scales between positive and negative feedbacks. For instance, in Mussel Beds, mussels will attach to rock aided by con-specifics that provide shelter, anchorage and nutrition^8,24^. Negative feedback occurs due to mussels competing for resources that are depleted over a longer scale: In the presence of a current, the directionality of the flow produces a shadow of competition down-flow of a mussel patch. Other similar mechanisms occur for semi-arid ecosystems, where local positive feedback occurs through sheltering and root structure and longer-range competition occurs due to depletion of ground water. Again directionality can occur due to sloping ground, meaning ground water will flow in one direction^9,25–28^. Finally Seagrass also exhibits local and long-range feedbacks. Short-range positive feedback is due to sheltering and nutrient deposition. The long range negative interaction this time comes from hydrological scouring, where in places with strong current water flow scours the ground reducing the probability of young shoot growth down flow of a high density area of seagrass^29–31^.

**Figure 1:**
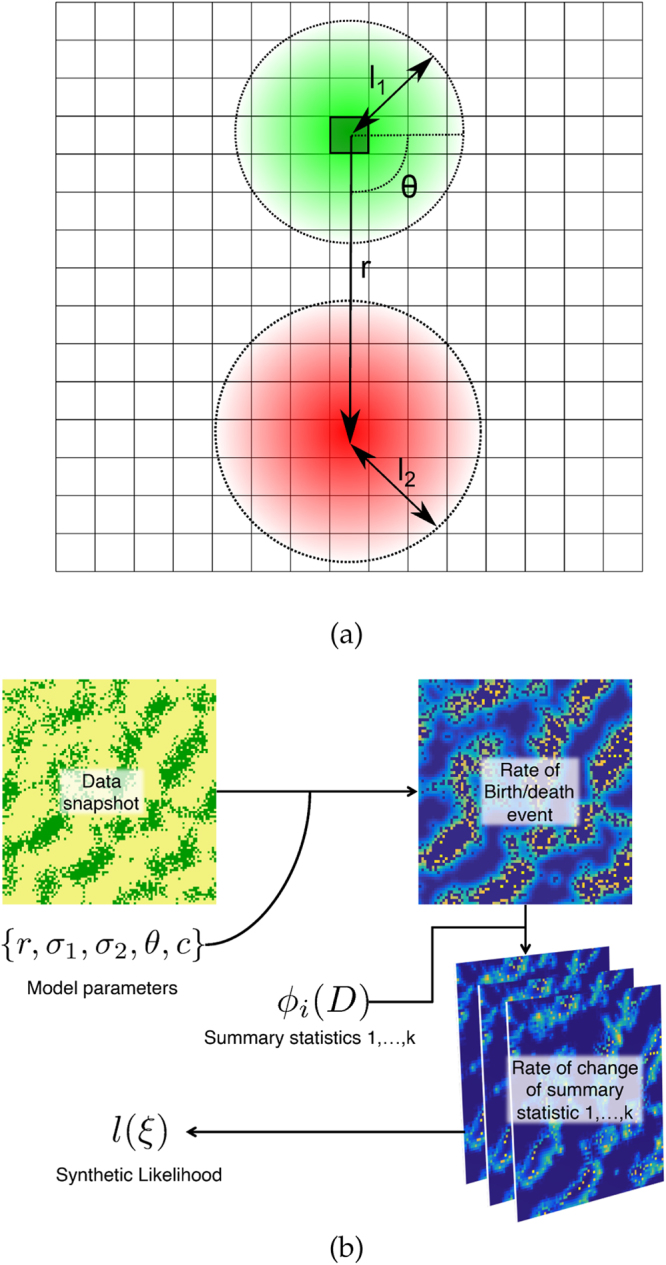
(**a**) Schematic of PCA model. An occupied site is shown as a dark square with border, with its kernel of reproduction surrounding it with length-scale ${l}_{1}={\sigma }_{1}^{2}$. The competition kernel is at angle *θ* and displacement *r*, with its own characteristic length-scale ${l}_{2}={\sigma }_{2}^{2}$. **(b)** Overview of likelihood generation. Procedure begins with a set of model parameters and the underlying data (top-left). Using the model-based rates, the rate of a birth/death event can be calculated for each lattice site (top-right). A choice of summary statistics can then be applied to these rates to produce a set of rates of changes of summary statistic for each lattice site, which is then summed over the whole lattice (bottom-right). These are then aggregated together to produce a single value, which represents the synthetic likelihood *l*(*ξ*).

In order to model these mechanisms we consider a probabilistic cellular automata (PCA) on a lattice with periodic boundary conditions. PCA with long-range dynamics have been used previously to model vegetative processes^32–34^. Each site in the lattice (*S*) is denoted (*i*, *j*) and can either be occupied (1) or unoccupied (0). The dynamics are updated synchronously and the probability at which an unoccupied site becomes occupied is proportional to the local density of occupied sites (locality is defined via a local growth kernel *k*_1_, which is Gaussian with variance ${\sigma }_{1}^{2}$). Death is assumed to be due to over-crowding and is mediated via a competition kernel *k*_2_, which is also assumed to be Gaussian, but locally offset (See Fig. 1a for a schematic overview). Hence the transition rates for an individual site (*i*, *j*) are

2a $${\rm{Birthrate}}:{r}_{1}(i,j)=\lambda ({[{k}_{1}\ast S]}_{ij}),$$

2b $${\rm{Deathrate}}:{r}_{0}(i,j)=c({[{k}_{2}\ast S]}_{ij}),$$

where * is the standard two-dimensional convolution operator defined as ${[u\ast v]}_{ij}={\sum }_{k}{\sum }_{l}{u}_{i,j}{v}_{i-k,j-l}$. *λ* is the rate of reproduction. As we consider only a single snapshot, time may be arbitrarily rescaled. We do so by setting *λ* equal to one, thus effectively removing a parameter from the model. Space is also similarly scaled such that lattice squares are 1 × 1 units. *c* controls the strength of the competition, where a low value of *c* reduces the overall death rate due to competition. It is also assumed that there is some anisotropy to the competition; as such, the *k*_2_ kernel is offset by distance *r* and angle *θ*. The parameters of the model are summarised in Table 1.

**Table 1:** Summary of model parameters.

Parameter	Description
*λ*	birth rate (set to one unless otherwise stated)
${\sigma }_{1}^{2}$	variance of growth kernel
${\sigma }_{2}^{2}$	variance of competition kernel
*c*	strength of competition
*θ*	direction of competition kernel offset
*r*	displacement of competition kernel offset

### Data

The data were collected via remote sensing techniques. The Seagrass data were collected in the form of an aerial photographic survey conducted in the Isles of Scilly, UK (49°55′N, 06°19′W) during the summer of 2008^35^. Meadow locations were surveyed and the resulting images were digitised and converted into a binary lattice. This was achieved through a combination of image analysis, ground truthing and historical data mining. The aerial photographs were pre-processed to mask out land and build a mosaic of the separate images. Unsupervised classification of the processed images was then carried out on the red, blue and green bands to objectively classify pixels into categories containing similar values for each of the three variables. Ground truthing was then used to determine which clusters were seagrass (see Jackson *et al*.^35^ for a full overview of the method).

We selected two regions where different forms of banding are exhibited. Regions of spatial patterning were located at (49°57.588′N, 6°18.578′W) (snapshot A) and (49°57.467′N, 6°21.362′W) (snapshot B) in 100 × 100 m spatial snapshots. Each aerial photograph was digitized to produce two binary lattices representing the occupation of seagrass in the site^35^. Snapshot A contains an area of strong sharp bands and snapshot B is a region where there is little to no banding.

Synthetic data were generated from the model. A lattice of size 100 × 100 was initiliased randomly with 10% occupancy. The system was allowed to evolve for 1000 time-steps until the population size and pair-wise correlations reached statistical equilibrium. The parameters $r=\mathrm{10,}\,\theta =\mathrm{1.5,}\,{\sigma }_{1}=\mathrm{0.6,}\,{\sigma }_{2}=\mathrm{2,}\,c=1$ were chosen from a region of parameter space where strong banding occurs.

### Overview of Fitting Process

The fitting process can be divided into two parts: the construction of the synthetic likelihood and the implementation of this likelihood to generate well-fitting parameters. An overview of the likelihood construction is given in Fig. 1b.

Construction of the likelihood is performed on data, *D*, composed of a single spatial snapshot composed of a lattice of sites that are either occupied or unoccupied (top-left of Fig. 1b). The birth and death rates at each site are calculated for the whole lattice based on a particular set of model parameters Θ based on the rates described in Eq. 2 (Top-right of Fig. 1b). Using these statistics, the expected rate of change in the pair-wise correlations at a distance *d* are calculated for *d* = 1, …, *k* (Bottom-right of Fig. 1b). Finally these values are input into a synthetic likelihood function that can be used for model fitting (bottom-left of Fig. 1b).

The outline for the methodology is to first provide a calculation of the expected rate of change at each lattice site, which is then used to construct the pair-wise expected rate of change for a range of pair distances. A likelihood is then constructed based on these summary statistics and finally this is incorporated into a Bayesian fitting procedure. This is summarised in Alg. 1 and the details of each step are outlined below.

**Algorithm 1:**
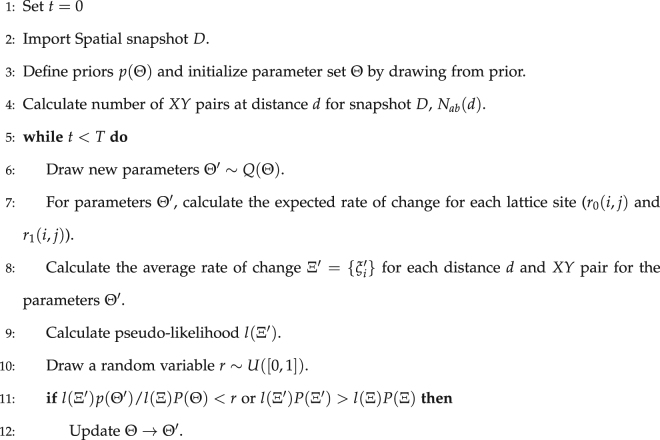
Fitting procedure using correlation-based pseudo-likelihood.

### Calculation of pair-wise correlations

We use the pair-wise correlations between sites at distance *d*, denoted *P*_*ab*_(*d*) as the main statistics in the construction of the synthetic likelihood. The correlations occupied-occupied, unoccupied-occupied and unoccupied-unoccupied were all used. The distance metric used was Euclidean, i.e. $d=\sqrt{{x}^{2}+{y}^{2}}$, where *x* and *y* are the longitudinal and latitudinal displacement defined on the lattice. For a site (*i*, *j*) in state *X*, the number of sites in state *Y* at distance *d* away is ${N}_{XY}^{(i,j)}(d)$. Unlike in the model simulations, periodic boundary conditions cannot be assumed. We therefore introduce a boundary around the lattice, where sites are not directly considered (See Supplementary information). The pair-wise count was then normalised over the total number of lattice sites at distance *d* away, *N*(*d*). Note that these correlations do not depend upon the model parameters and only need to be calculated once for each lattice data (Step 4 in Alg. 1).

### Calculation of the expected rate of change

The data *D* is a spatial binary (1 for occupancy, 0 for empty) lattice indexed by (*i*, *j*). For each site (*i*, *j*) in the lattice and a given set of parameters, the model birth rate *r*_0_(*i*, *j*) and death rate *r*_1_(*i*, *j*) can be calculated dependent on whether the site is occupied or unoccupied (step 7 in Alg. 1), these values are then combined with the number of pairs at distance *d* described in the following step.

### Calculation of expected change in *XY* pairs at distance *d*

For a given set of model parameters Θ = (*λ*, *σ*_1_, *σ*_2_, *c*, *r*, θ), the expected rate of change in each *XY* pair at distance *d* is calculated using the rate of a birth event and death event at every lattice site. We only consider first-order transitions where at most one lattice site changes. Each birth and death event contributes to both the creation and destruction of pairs 00, 01 and 11 and hence must be considered for each site. As an example consider the expected change in correlations for the pair 00 at distance *d* due to an event at site (*i*, *j*). A 00 pair is created either due to a death at that site (which occurs at rate *r*_0_(*i*, *j*)) multiplied by all the 01 pairs at distance *d* for that site, ${N}_{10}^{(i,j)}(d)$. Similarly the expected number of 00 pairs lost is the rate of a birth at that site (*r*_1_(*i*, *j*)) multiplied by the number of sites that are unoccupied at distance d from that site, ${N}_{00}^{(i,j)}(d)$.

3 $${\rm{\Delta }}{r}_{00}(d)(i,j)={r}_{0}(i,j){N}_{10}^{(i,j)}(d)-{r}_{1}(i,j){N}_{00}^{(i,j)}(d\mathrm{).}$$

This is then summed over all sites (*i*, *j*) to calculate the expected rate of change of *XY* pairs at distance *d* (step 8 in Alg. 1). In order to remove boundary effects, only lattice sites within a given boundary are summed over as sites on the edge of any snap-shot are affected by sites that are unobserved. This produces a set of summary statistics for each distance *d* and *XY* pair (we denote this using ${\rm{\Xi }}=\{{\xi }_{i}\}$ where *i* is an index over *XY* pairs and distances *d*) (step 8 in Alg. 1). A derivation for each pair is given in the Supplementary information (Derivation of expected change of *XY* pairs).

### Calculation of synthetic likelihood

Using collection of statistics, Ξ, we construct a probability distribution for each *XY* pair and distance *d*. The Likelihood was then derived by assuming that each statistic ${\rm{\Xi }}=\{{\xi }_{i}\}$ is independent and has normal error with variance ${\tau }_{i}^{2}$, hence producing the resulting likelihood

4 $$l({\rm{\Xi }})=\prod _{i=1}^{N}\frac{1}{\sqrt{2\pi {\tau }_{i}^{2}}}\,\exp \,(-\frac{1}{2{\tau }_{i}^{2}}{\xi }_{i}^{2}),$$

where *i* sums over all pairs (00, 01 and 11) and all distances (1, …, *k*) (step 9 in Alg. 1). The log-likelihood function is

5 $$\mathrm{log}\,l({\rm{\Xi }})=-\sum _{i=1}^{N}(\frac{1}{2}\,\mathrm{log}\,\mathrm{(2}\pi {\tau }_{i}^{2})+\frac{{\xi }_{i}^{2}}{2{\tau }_{i}^{2}})\mathrm{.}$$

**Figure 2:**
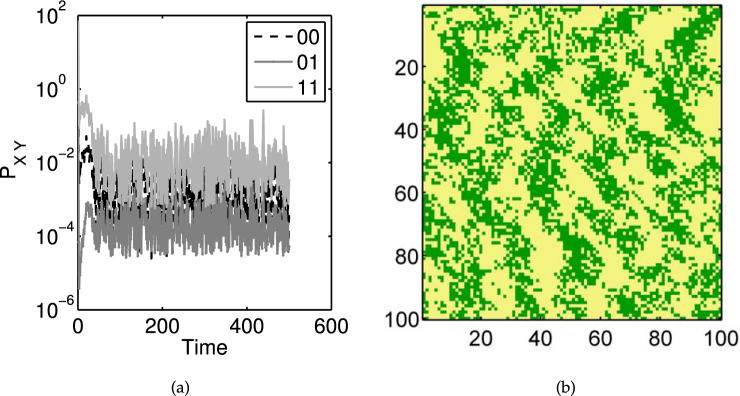
(**a**) The individual errors for each of the correlation observables for a single simulation run. The system quickly relaxes to a statistically stationary state where the size of the errors for each observable are comparable to one another. **(b)** Example simulation of banding pattern at statistical stationarity on a 100 × 100 lattice with periodic boundary conditions. Patches of vegetation coalesce together to form bands where locally their orientation changes, but are all produced from the same values of *r* and *θ*.

We found through simulation over a range of lattice sizes and parameters that the normal distribution characterized the distribution of the observables, although other distributions such a the exponential distribution could be considered depending on the model and observables that are studied. There is still an issue over the value of ${\tau }_{i}^{2}$ for each observable *i*. It was found from typical simulation runs that the variance scales with the number of pairs as a square. Hence, the functional form of ${\tau }_{i}^{2}(d)={\tau }^{2}N{(d)}^{2}$ for pairs 00, 11 and 01 (Fig. 2a). The functional form of the variance then leaves a single free parameter for the likelihood, which can then be found through simulation over a range of parameters. For a 100 × 100 lattice we derived a variance of ${\tau }^{2}={10}^{-6}$.

### Fitting procedure

In order to fit the model likelihood to a given dataset a Metropolis-Hastings Markov Chain Monte Carlo (MH MCMC) sampling scheme was implemented, where the posterior may be sampled (note that this is distinct from the Markov chain underlying the model. See Supplementary information for a derivation). Initially the parameters are drawn independently from their prior distribution ${\rm{\Theta }}^{\prime} \sim p({\rm{\Theta }})$ (step 3 in Alg. 1). A proposal parameter was then chosen from a normal distribution centred around the current parameter, with the transition probability denoted as *Q*(Θ) (step 6 in Alg. 1). The proposal is then accepted according to the probability

6 $$\alpha =\,{\rm{\min }}\{\mathrm{1,}\frac{l({\rm{\Theta }}^{\prime} )p({\rm{\Theta }}^{\prime} )}{l({\rm{\Theta }})p({\rm{\Theta }})}\},$$

which corresponds to step 11 in Alg. 1. As the transition is a symmetric random walk we can ignore the transition probability in the ratio term. We chose the transition probabilities to be independent for each parameter and normal centred on the current parameter value. The variance for each transition distribution was tuned during the burn-in phase to produce an acceptance rate of approximately 25% for each parameter^36^. Experimentation of the acceptance rate supported the choice of 25% acceptance providing a good compromise between efficiency and mixing of the chain. The posterior was sampled from the MCMC chain after a burn-in of 10^4^ steps and 10^6^ sampling steps. With consideration to the acceptance rate, thinning was not performed^37^. Visual inspections of the chains indicated sufficient mixing during the sampling and convergence during the burn-in period^38^.

### Choice of priors

In order to regularise the region of parameter space over which fitting occurs, empirical weak priors for the spatial parameters were formulated from the lattice spatial data. An exponential prior for the displacement *r* was chosen, with a mean corresponding to the average inter-band width. Gamma distributions were generated for the variances of the reproduction and competition kernels with means of the average band width and inter-band gap width respectively. The variance of the gamma distributions were chosen to be sufficiently wide (10% the length of the lattice). Although these priors are informed by the data, they are not informed from the model and are there to weakly constrained the parameters to a biologically realistic region of parameter space. The prior and posterior distributions still displayed strong differences demonstrating the likelihood was informative. Further the competition parameter *c* was given an exponential prior with a mean 1 and the direction of the competition offset was uniform on the interval [0,π] for all data considered in the study.

### Scenario analyses

#### Model simulations

The model was explored for a range of parameters in order to understand the impact of each parameter on the formation of spatial patterns. Simulations were ran for up to 1000 time-steps until spatial patterns stabilized. In order to understand how the summary statistics evolve in time and to determine the scaling in system size the expected change in pair-wise correlations at distance *d* were recorded for a number of parameters and system sizes from 50 × 50 to 1000 × 1000 and for 500 time-steps. We used the Kolmogorov-Smirnov test^39^ to determine whether the correlations produced from the model simulations deviated significantly from a normal distribution.

#### Fitting to simulated data

The fitting procedure was carried out on a region of parameter space known to produce strong banding ($r=\mathrm{10,}\,\theta =\pi \mathrm{/2,}\,{\sigma }_{1}=\mathrm{0.6,}\,{\sigma }_{2}=\mathrm{2,}\,c=1$). This region was determined through grid search, where angle was arbitrarily set to *π*/2. Although the region of banding is quite broad in parameter space, banding collapses where the displacement of competition (*r*) is too low or the reproduction or competition kernel widths (*σ*_1_ and *σ*_2_) are too large (see Supplementary information). The inferred parameters were then compared to the true underlying parameters in order to assess if the original parameters could be correctly determined.

#### Fitting to real data

The fitting procedure was then carried out on the two seagrass meadow spatial data. The marginal posteriors were compared in order to assess how the disparate spatial patterns were resolved by the parameters.

### Data availability

Both Matlab and Python libraries for the described fitting technique can be found at the following url, https://github.com/sempwn/spatial-inference.

## Results

### Model simulations

The model produces banded structures, with regular regions of high density and regions of low density at regular intervals. Each parameter has a different impact on the observed patterning of the banding. The competition parameter *c* controls the strength of the banding as well as the visual sharpness of the boundaries. When $c\ll 1$, competition is low and hence the strength of banding is weak with rough boundaries. For *c* > 1, bands are much sharper and the inter-band gaps have a high density of unoccupied states. The length-scale of the local growth *σ*_1_ shows strong interaction with the length-scale of the competition *σ*_2_ (Fig. A2 in Supplementary Information). Both interact to produce the characteristic length-scale of the bands. Also the length-scale of the bands and the inter-band gap show strong correlation and are of the same order of magnitude across all range of parameters. *r* is defined as the displacement of the offset for the competition. If *r* is too small compared to the length scale *σ*_1_, then competition overwhelms the local growth and thus the whole population goes extinct. There therefore exists a minimum *r* after which banding will occur. Lastly *θ* gives the orientation of the offset for the banding and as such bands form in a perpendicular direction to the orientation of the competition.

Frustration was also observed in the banding of the system. This is where there exists a topological defect in the banded structure of the emergent spatial pattern. A topological defect is defined as when a contour can be drawn around the defect in such a way that the number of bands on one side of the contour do not equal the bands on the other side of the contour. As such the bands will not align without the destruction or creation of new bands. This leads to the situation where the overall correlation structure does not change over time, but fluctuations in the correlations occur as a defect moves through the lattice.

Simulations were performed for a number of system sizes and parameter values drawn randomly in order to determine the scaling of the errors as the size of the lattice increases. As the simulation is run the ${P}_{00}^{2}(d)$, ${P}_{01}^{2}(d)$ and ${P}_{11}^{2}(d)$ errors move through a transient state and quickly relax leading to smaller fluctuations compared to the transient state, where this was noted through visual inspection of the error time-series (Fig. 2a). The statistic *ζ* ^2^ was recorded over a number of time steps and for increasing system sizes. Using the Kolmogorov-Smirnov test^39^, it was found that all the correlation errors did not reject the null hypothesis that they were drawn from a normal distribution at level α = 0.05 and hence the assumption that the errors are normally distributed has evidence that it is correct.

### Fitting to synthetic data

As an example of using the technique to fit to binary vegetation data, a simulation was performed on a 100 × 100 grid with parameters $r=\mathrm{10,}\,\theta =\pi \mathrm{/2,}\,{\sigma }_{1}=\mathrm{0.6,}\,{\sigma }_{2}=\mathrm{2,}\,c=1$ and periodic boundary conditions. The parameters were chosen to be in a region where strong banding occurs and hence is ideal to test how well the technique performs (Fig. 2b).

Although the offset is constant throughout the simulation the local banding structure of the simulation has a varying orientation for different regions of the lattice. Frustration also occurs, where clusters are not aligned with neighbouring bands (See (60, 60) co-ordinates in Fig. 2b for an example of this).

**Figure 3:**
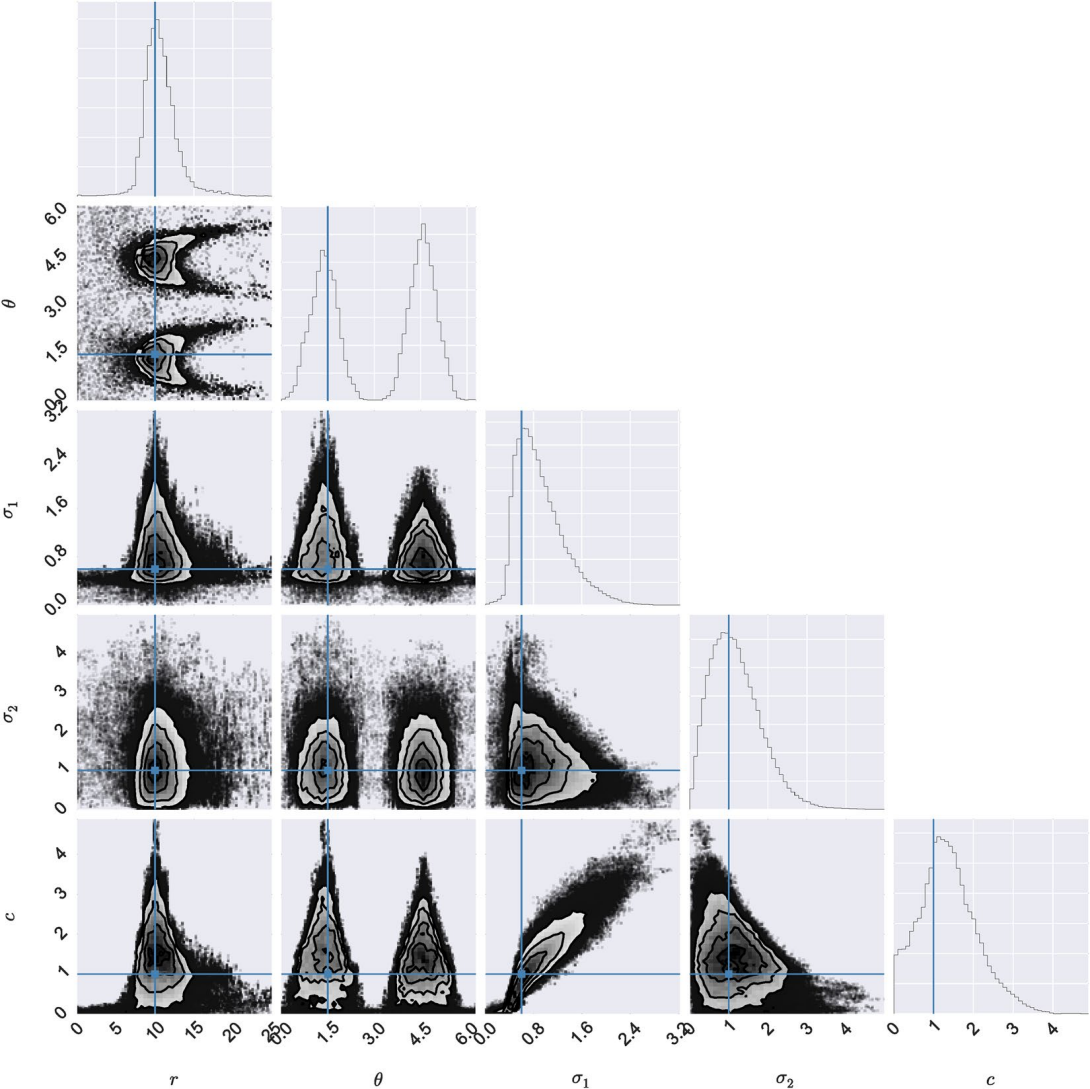
Example of posterior for a 100 × 100 spatial snapshot ran until equilibrium with parameters $r=\mathrm{10,}\,\theta =\mathrm{1.5,}\,{\sigma }_{1}=\mathrm{0.6,}\,{\sigma }_{2}=\mathrm{2,}\,c=1$ (simulated data shown in Fig. 2b). The calculated marginal posterior for each parameter is shown, with true parameters as a blue solid line. The posterior correlation structure is given with a contour plot and 95% outliers as black dots. For all parameters the posterior is within 95% confidence intervals of the true parameters.

The posterior was estimated using a MH MCMC scheme run for 1 × 10^6^ time-steps after a sufficient burn-in time. There is a bimodal relationship between the *r* and *θ* parameters producing two peaks at *θ* ≈ 1.5 and *θ* ≈ *π* + 1.5 (See Fig. 3) where the highest density of the posterior is contained. This gives a mode for *r* of around 10 (7.8–14.9 95% credible interval (CI)). The mode of the marginal distributions for each of the parameters falls close to the true values from the simulation. In particular the true value is contained in each of the 95% probability densities for each of the marginal distributions. The local growth width *σ*_1_ (0.4–2.0 95% CI) has an approximately gamma distribution with mode less than the approximate gamma distribution of *σ*_2_ (0.4–3.0 95% CI). Thus the marginals of *σ*_1_ and *σ*_2_ give a way of testing the hypothesis that the extent of the competition is greater than that of the extent of the local growth i.e. *σ*_1_ < *σ*_2_.

### Fitting to real data

**Figure 4:**
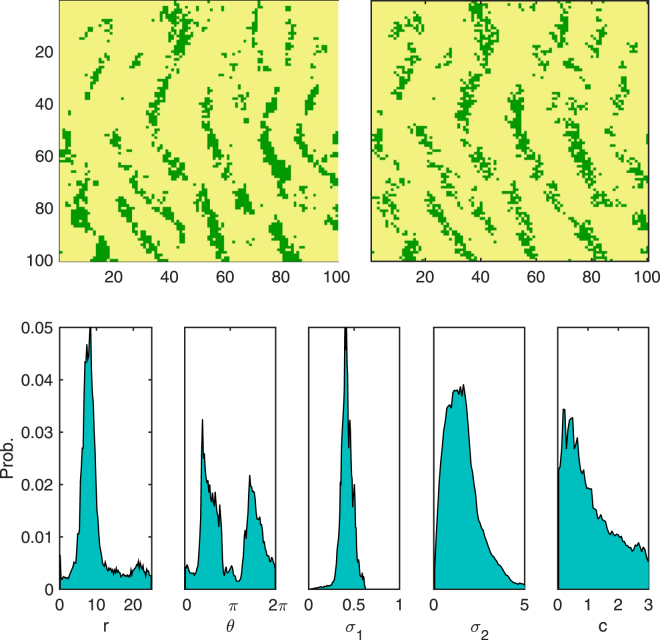
Top-left: Snapshot A: Example of strong banding pattern observed in seagrass. Top-right: an example simulation with parameters drawn from posterior distribution and the original lattice used as initial conditions. Bottom: marginal probability distribution for each parameter.

**Figure 5:**
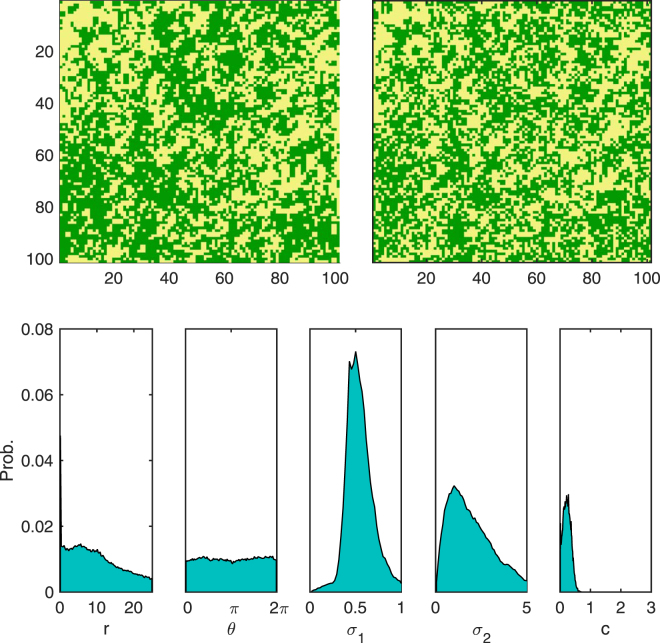
Top-left: Snapshot B: example of weak banding observed in seagrass. Top-right: an example simulation with parameters drawn from posterior distribution. Bottom: marginal probability distribution for each parameter.

With the validity of the scheme established we can now turn our attention to real data in the form of an aerial photographic survey of seagrass. We select two regions where different forms of banding are exhibited. Regions of spatial patterning were located at (49°57.588′N, 6°18.578′W) (snapshot A) and (49°57.467′N, 6°21.362′W) (snapshot B) in 100 × 100 m spatial snapshots. Each aerial photograph was digitized to produce two binary lattices representing the occupation of seagrass in the site^35^. Snapshot A contains an area of strong sharp bands (Fig. 4). This is reflected in the marginal posterior, where there is a sharp peak in *r* around 10 m with a sharp bimodal structure of *θ* with peaks at approximately *π*/2 and 3*π*/2. The width of the reproduction (*σ*_1_) is sharply defined with a peak at 0.5. The banded structure is also reflected in the posterior of the competition *c*, where the marginal has a long tail with a mode of approximately 1. Finally we look at snapshot B, a region where there is little to no banding (Fig. 5). By contrast to the sharp banded structure of snapshot A, the marginal posterior of *r* occupies a large range, with approximately constant probability between 0–10 m. The orientation of the competition is approximately uniform with a negligible increase around *π*/4. The local reproduction width meanwhile is sharply defined with peak between 0.75–1. There is also a much lower predicted value of *c*, indicating less spatially aggregated competition.

## Discussion

In this paper we have derived a novel method of inferring parameters of a probabilistic cellular automata model based upon the expected rate of change in the observed spatial pattern. The main idea is to infer dynamic parameters from a snapshot alone. We have greatly extended the work of Keeling *et al*.^15^ by taking the concept of using the expected rate of change of pair-wise correlations to construct a synthetic likelihood. This likelihood was then used to develop a full Bayesian methodology and applied to a model of vegetation with spatial competition. It was found that when the banding pattern is strong, parameters are well-informed by the synthetic likelihood, however there is a high amount of uncertainty when banding in the spatial snapshot is weak or non-existent.

The probabilisitic cellular automata model was able to qualitatively reproduce banding observed in nature. The underlying mechanism for this was based on local facilitation due to a local reproduction kernel with a fixed length and an offset competition kernel with a separate characteristic length scale. There are, however, many other known mechanisms that can induce pattern formation in sessile organisms^23^. Therefore, a strong understanding of the system is necessary in order to correctly choose an applicable model. In the case of seagrass, it is known that the effects of currents and waves induce spatial competition^40^. This implies there is a partially validated mechanism for inducing pattern formation that supports the choice of model. Another assumption of the model is landscape homogeneity. This is applicable on smaller spatial scales (100 m), but would begin to break down on larger scales as local currents and sea depth varies. In order to incorporate larger-scale data into the model and fitting scheme a hierarchical approach could be used, where the parameters are spatially-varying with a spatial correlation structure that can be inferred^41^. The competition strength *c* is less well-defined, although the marginals do vary as the underlying value of *c* is varied. This may be due to the issue that the transition between no banding and banding is sharp and hence the correlation structure may only be able to distinguish between when *c* is small or large. The reproduction and competition variance, *σ*_1_ and *σ*_2_ have well-defined peaks for smaller values, but these are less well defined when both variances are larger. This corresponds to the break down of banding and thus strong correlation structure as the variance in reproduction or competition grows (See Fig. A2 in Supplementary Information for a full analysis of *σ*_1_ and *σ*_2_).

Previous studies have considered deriving the strength of facilitation and competition from a spatial snapshot using a PCA model^42,43^. Our contribution here is to construct a quantitative method for deriving these parameters from a snapshot, including the full uncertainty in these parameters. The method also produces a set of parameters from which scenarios (such as a storm disturbance, see the supplementary information) may be simulated, providing uncertainty around the outcome.

The idea behind the synthetic likelihood is instead of considering the full likelihood (which would likely be intractable or computationally prohibitive to implement), we instead consider a likelihood built from a set of summary statistics. The summary statistics developed were the expected rate of change of pair-wise correlations. The advantage of this approach is that a likelihood can be described for a single spatial snapshot and only the model rates need to be calculated at each iteration. It is however, an approximation to the full likelihood and is dependent on the summary statistics fully describing the current dynamic state. This is why the model fit becomes more resolved when there is a strong spatial pattern (in terms of the pair-wise correlation structure), compared to when there is no discernible pattern.

We performed Bayesian inference for the study. This involves defining a prior for the parameters used in model fitting and sampling from a posterior, which is a combination of both these and the likelihood. The prior can help to regulate fitting as well as be used to include any other information known about the system. For instance if it is known over what length-scales competition acts, this can be included as a prior. In order to determine the prior here, the inter-band, between band and band length were all measured to produce weakly informed-priors. This was done in order to constrain where competition acts to the nearest neighbour band. If this was relaxed, such as through the use of improper priors, the marginal posterior for the competition displacement would become multi-modal. This choice of prior therefore constrains the parameters to the biologically-realistic regime.

The method can be extended in a number of ways. We have investigated the phenomena of banding for a single vegetation ecosystem, the kernel model is quite general however and can be applied to other processes such as other types of regular patterns as well as disease. For such systems the evolution of the spatial pattern would have to be slow, such that transients in the correlation functions should quickly die out. The approach should also work for spatio-temporal data, where several snapshots through time exist for a specific location in order to detect change between snapshots. The increased number of snapshots could easily be fed into the method and used to more accurately infer model parameters without inferring the behaviour between snapshots. The pair-wise correlations can also be extended to consider triplets i.e. X-Y-Z correlations. It would be interesting to measure how this extension would perform in an inference task. The likelihood may also include an uncorrelated noise term in order to account for inaccuracies in the image classification.

More realistic asymmetric reproduction and competition kernels could readily be adapted into our scheme. We have found, however that there is some difficulty in inferring the exact shape of the competition kernel beyond variance(results not shown). Nevertheless, more realistic kernels could be implemented if their distributions were known (e.g. through the use of flume experiments in the case of seagrass^44^).

Other methods that could be applicable for inference on spatial pattern would be *Approximate Bayesian Computation* ^45^. This however, would require many simulation runs and for a large system size could potentially be computationally costly. The method described is able to perform inference without the need for simulation or continued calculation of the spatial correlation functions. Inference of anisotropic parameters using a model-based approach has advantages over other approaches such as spectral methods, where parameters cannot be related back to a PCA model. With a correctly tuned model, various scenarios such as temperature increases or disturbances of the environment can be explored and hence can help to inform ecosystem management under certain scenarios.

Other systems where the model is applicable include mussel beds and tiger bush. Mussel beds have been shown to self-organise into regular banded patterns^8^. Their local interaction is due to the reduction of dislodgement and the reduction is predation as the density of mussels in the local area increases. Long-range competition is induced through the depletion of algae, that mussels prey upon, due to the presence of mussels depleting the resource on a long-range scale. For our model, there must be the assumption that this depletion has a preferred direction, due to prevailing currents. Semi-arid ecosystems can also develop banded patterns known as Tiger Bush. The principles of local positive long-range negative feedback are similar to the previous two cases. Local positive feedback occurs via uptake of ground water, which is shared among plants in the local area. Shadowing and sheltering can also help to increase ground water above mean rates. the presence of high density at longer range means there’s a depletion of ground water and thus a lack of resources needed for growth leads to new plant colonies being unable to establish. Striped patterns can occur where the environment is homogeneous, but for our purposes we consider the formation of stripes due to an environmental gradient, such as sloping. This induces a preferred direction to the long-range competition, thus making the model applicable. It is interesting to note that two main ingredients seem to reproduce the wide-variety of pattern observations found in vegetation and sessile communities: separation of scale for feedbacks and noise.

## Conclusion

We described a novel method for probabilistic cellular automata model fitting to a spatial snapshot of vegetation occupancy. The method described was capable of reproducing model parameters for synthetic data and able to reproduce qualitative spatial pattern for two seagrass meadows with different spatial patterns. This represents a promising step towards inferring dynamic information from a single spatial snapshot.

## Electronic supplementary material

Supplementary information

LaTeX Supplementary File
