# Supplementary material for: Conservation of pattern as a tool for inference on spatial snapshots in ecological data
Source: Sci Rep. 2018 Jan 9;8:132. doi: 10.1038/s41598-017-17346-6 (PMC5760736; doi:10.1038/s41598-017-17346-6)
Supplement: Supplementary file 1 — Supplementary information [file 41598_2017_17346_MOESM1_ESM.pdf]

Conservation of pattern as a tool for inference on spatial snapshots in ecological data: Supplementary Information

Michael A Irvine[1,∗]

James C Bull[2,†]

Matt J Keeling[1,3,‡]

1. Institute of Applied Mathematics, University of British Columbia, Vancouver, V6T 1Z2, Canada;

2. Department of Biosciences, Wallace Building, Swansea University, Swansea, SA2 8PP, UK;

3. Mathematics Institute, University of Warwick, Coventry CV47AL, UK.

∗ Corresponding author; e-mail: m.irvine@math.ubc.ca

† e-mail: j.c.bull@swansea.ac.uk

‡ e-mail: m.j.keeling@warwick.ac.uk

Appendix A: Supplementary Figures

Marginal posterior with altering parameters

In order to probe impact on varying each parameter of the model from the test spatial snapshot (where $r = 10, \theta = \pi/2, \sigma_1 = 0.6, \sigma_2 = 2, c = 1$), each parameter in turn was altered to produce a new spatial snapshot from random initial conditions. The synthetic likelihood-based inference was then performed on each of these snapshots in turn, where the priors were kept the same throughout, and the marginal posteriors of the parameters that had been varied from the baseline were recorded (Fig. A1). Changing each parameter significantly altered the corresponding marginal posterior. r and θ were particularly distinct, where the peak of the marginal shifted to the corresponding new value of r and θ. For increasing r, the peak of the distribution reduces corresponding to a decrease in the certainty of the offset of the competition. The θ distributions are bimodal, where one peak corresponds with the angle of the offset and the other peak corresponds to the offset in the opposite direction. The competition strength c is less well-defined, although the marginals do vary as the underlying value of c is varied. This may be due to the issue that the transition between no banding and banding is sharp and hence the correlation structure may only be able to distinguish between when c is small or large. The reproduction and competition variance, σ_1 and σ_2 have well-defined peaks for smaller values, but these are less well defined when both variances are larger. This corresponds to the break down of banding and thus strong correlation structure as the variance in reproduction or competition grows as can be seen in Fig. A2

Appendix B: Disturbance Simulations

We explored the impact of the fitted parameters on the resilience of the system by simulating a disturbance event such as from storm damage. Maximum a posteriori model parameters were selected with random initial starting conditions and the simulation was ran for 1000 time-steps until the population size had reached equilibrium. A disturbance event was then simulated by removing 50% of the population uniformly at random. The return to pre-disturbance population size was then measured. Note that these measurements are in arbitrary time-units due to the re-scaling the the growth rate λ.

Results

We considered specific scenarios occurring for each example snapshot. The maximum a posteriori were taken and simulations were run until the total density reached stationarity. A uniform disturbance was then applied whereby 90% of the occupied sites were removed at random. The density time-series was then recorded for both snapshots. There is a marked difference in the recovery characteristics between the two sites (Fig. B1). Snapshot B, where there is less spatial competition, has a faster recovery to a higher density than snapshot A. The time to the carrying capacity of the population was similar for both, however.

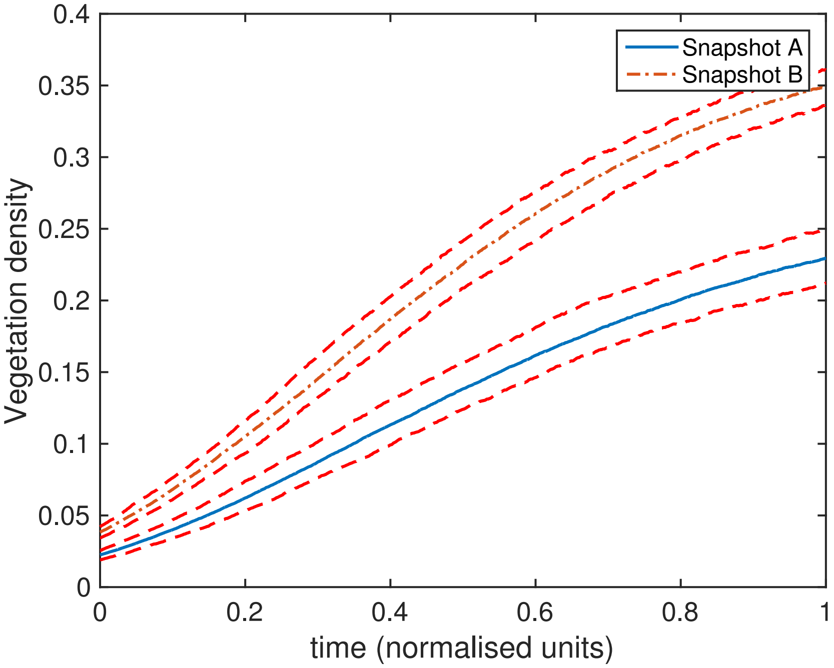

Figure B1: Example simulations from *maximum a posteriori* parameters inferred from each example snapshot. A uniform disturbance that removes 90% of the vegetation is simulated and the subsequent density time-series is plotted. Snapshot B, where there is less spatial competition has a faster recovery and higher density than snapshot A.

Appendix C: Additional Methods

Derivation of the variational correlation for spatial pattern inference

We wish to derive the full term of the estimator used in the spatial correlation inference. We begin by assuming the Kolmogorov forward equation for the probability of a system to be in state $S \in \Omega$. Given a rate of moving from state $S \to S'$ as $w_\Theta(S'|S)$, where $\Theta = \{\theta_1, \theta_2, \ldots, \theta_N\}$ are the parameters for the system,

$$\frac{\partial}{\partial t} P_t(S) = \int_\Omega \left[w_\Theta(S|S') P_t(S') - w_\Theta(S'|S) P_t(S) \right] dS'. \tag{C1}$$

We have some observable of the system state $\phi : \Omega \to \mathbb{R}$. For our particular example we have used spatial correlation as the observable, however this method is general and would apply to

any observable of the system. The expectation of an observable is defined as

$$\mathbb{E}[\phi(S)] = \int_{\Omega} \phi(S) P(S) dS. \tag{C2}$$

The rate of change of this expectation can then be calculated using Eq. C1

$$\frac{\partial}{\partial t}\mathbb{E}[\phi(S)] = \frac{\partial}{\partial t}\int_{\Omega} \phi(S) P_t(S) dS \tag{C3}$$

$$= \int_{\Omega} \phi(S) \frac{\partial}{\partial t} P_t(S) dS \tag{C4}$$

$$= \iint\limits_{\Omega\times\Omega} \phi(S) \left[w_{\Theta}(S|S')P_t(S') - w_{\Theta}(S'|S)P_t(S)) \right] dS' dS. \tag{C5}$$

Observe that

$$\iint\limits_{\Omega\times\Omega} \phi(S) \left[w_{\Theta}(S|S')P_t(S') - w_{\Theta}(S'|S)P_t(S)) \right] dS' dS \tag{C6}$$

$$= \iint\limits_{\Omega\times\Omega} \phi(S) w_{\Theta}(S|S') P_t(S') dS' dS - \iint\limits_{\Omega\times\Omega} \phi(S) w_{\Theta}(S'|S) P_t(S) dS' dS \tag{C7}$$

$$= \iint\limits_{\Omega\times\Omega} \phi(S) w_{\Theta}(S|S') P_t(S') dS' dS - \iint\limits_{\Omega\times\Omega} \phi(S') w_{\Theta}(S|S') P_t(S') dS' dS \tag{C8}$$

$$= \iint\limits_{\Omega\times\Omega} \left[\phi(S) - \phi(S') \right] w_{\Theta}(S|S') P_t(S') dS' dS. \tag{C9}$$

We define the change in an observable due to the transition to a new state to be $\Delta\phi(S|S') = [\phi(S) - \phi(S')]$. Hence we have

$$\frac{\partial}{\partial t}\mathbb{E}[\phi(S)] = \iint\limits_{\Omega\times\Omega} \Delta\phi(S|S') w_{\Theta}(S|S') P_t(S') dS' dS. \tag{C10}$$

We assume that the system is at statistical stationarity and so the expectation of the observable is time invariant. There is also a single observation of the system at equilibrium denoted E. The probability function of the state can then be estimated by a single delta function $\hat{P}(S) = \delta_E(S)$.

With the following assumptions we can then apply them to Eq. C10

$$0 = \iint\limits_{\Omega\times\Omega} \Delta\phi(S|S')w_\Theta(S|S')\hat{P}(S')dS'dS \tag{C11}$$

$$= \iint\limits_{\Omega\times\Omega} \Delta\phi(S|S')w_\Theta(S|S')\delta_E(S'))dS'dS \tag{C12}$$

$$= \int\limits_{\Omega} \Delta\phi(S|E)w_\Theta(S|E)dS. \tag{C13}$$

The estimator is therefore defined to be

$$\zeta_E(\Theta) = \int\limits_{\Omega} \Delta\phi(S|E)w_\Theta(S|E)dS. \tag{C14}$$

The integral currently goes over all possible states of the lattice $S \in \Omega$. For an $N \times N$ lattice size, this implies there are $2^{N\times N}$ possible combinations to sum over. In order to reduce the size of the integral to be performed we may approximate it by assuming that the transition rates of each site are small. It can then be assumed at each time step that the transitioning states are uncorrelated with one another and hence each state that E can transition to is $E(s)$ for all lattice sites s. $E(s)$ denotes the state E with site s switched. The integral now sums over all lattice sites s instead of all possible states and hence the size of the sum is $N \times N$ as opposed to $2^{N\times N}$. Hence, if I is the index set for the lattice S, $\zeta_E(\Theta)$ is approximated as

$$\zeta_E(\Theta) = \sum_{s\in\mathbb{I}} \Delta\phi(E(s)|E)w_\Theta(E(s)|E). \tag{C15}$$

Note that the transition rate $w_\Theta(E(s)|E)$ is the rate of site s transitioning, hence is either the rate of a birth at site s or the rate of a death at site s depending on the current state, hence we may simplify notation by defining $w_\Theta(s) := w_\Theta(E(s)|E)$.

Derivation of expected rate of change of XY pairs

The observables being considered are the pair-wise correlations at distance d. As seen in Eq. C15, each site contributes an amount towards the whole expected change in the observable. for any birth-death system there are three correlations to consider: 00, 01 and 11. The correlation

of 00 therefore is the probability that given a current site in state 0, a randomly selected site at distance d away is also in state 0 with probability $P_{00}(d)$. Eq. C15 is then used to calculate the expected rate of change of the observable $P_{00}(d)$. The increase or decrease of the observable for each site s depends on whether that site is occupied, in which case the only way in which the observable can change is if there is a death event at that site (at rate $d_\Theta(s)$) or when the site is unoccupied, in which case the only way the observable can change is if there is a birth event at that site (at rate $b_\Theta(s)$). Let $I(s)$ be the indicator function which is 1 if the site is occupied or 0 if it is unoccupied. The calculation of the rate of change of the observable $P_{00}(d)$ is therefore

$$\begin{aligned}
\frac{d}{dt}\mathbb{E}[\Delta P_{00}(d)] &= \sum_{s\in I} \Delta P_{00}(d)(E(s)|E)w_\Theta(s) \\
&= \sum_{s\in\mathbb{I}:I(s)=1} \Delta P_{00}(d)(E(s)|E)w_\Theta(s) + \sum_{s\in\mathbb{I}:I(s)=0} \Delta P_{00}(d)(E(s)|E)w_\Theta(s) \\
&= \sum_{s\in\mathbb{I}:I(s)=1} \Delta P_{00}(d)(E(s)|E)d_\Theta(s) + \sum_{s\in\mathbb{I}:I(s)=0} \Delta P_{00}(d)(E(s)|E)b_\Theta(s) \\
&= \sum_{s\in\mathbb{I}:I(s)=1} N^s_{10}(d)d_\Theta(s) - \sum_{s\in\mathbb{I}:I(s)=0} N^s_{00}(d)b_\Theta(s),
\end{aligned}$$

where $N^s_{XY}(d)$ represents the number of pairs where site s is in state X and the sites distance d away are in state Y. As an example, if site s is in state 0 and there is a birth event at site s then the number of 00 pairs that are destroyed at distance d apart due to this event is $N^s_{00}(d)$.

The expected rate of change for the pairs 01 and 11 at distance d apart may similarly be calculated by considering how each event at a site contributes to the creation or the destruction of a XY pair. The calculation for 01 is

$$\begin{aligned}
\frac{d}{dt}\mathbb{E}[\Delta P_{01}(d)] &= \sum_{s\in I} \Delta P_{01}(d)(E(s)|E)w_\Theta(s) \\
&= \sum_{s\in\mathbb{I}:I(s)=1} \Delta P_{01}(d)(E(s)|E)d_\Theta(s) + \sum_{s\in\mathbb{I}:I(s)=0} \Delta P_{01}(d)(E(s)|E)b_\Theta(s) \\
&= \sum_{s\in\mathbb{I}:I(s)=1} (N^s_{11}(d) - N^s_{10}(d))d_\Theta(s) + \sum_{s\in\mathbb{I}:I(s)=0} (N^s_{00}(d) - N^s_{01}(d))b_\Theta(s).
\end{aligned}$$

Similarly the calculation for the 11 pair is

$$
\begin{aligned}
\frac{d}{dt}\mathbb{E}[\Delta P_{11}(d)] &= \sum_{s\in I} \Delta P_{11}(d)(E(s)|E) w_{\Theta}(s) \\
&= \sum_{s\in\mathbb{I}:I(s)=1} \Delta P_{11}(d)(E(s)|E) d_{\Theta}(s) + \sum_{s\in\mathbb{I}:I(s)=0} \Delta P_{11}(d)(E(s)|E) b_{\Theta}(s) \\
&= -\sum_{s\in\mathbb{I}:I(s)=1} N^{s}_{11}(d) d_{\Theta}(s) + \sum_{s\in\mathbb{I}:I(s)=0} N^{s}_{01}(d) b_{\Theta}(s).
\end{aligned}
$$

We wish to calculate the total rate of change for a correlation pair XY for all distances. This is accomplished by constructing a weighted sum over all distances d, where the weight of each sum corresponds to the inverse of the number of sites at distance d from the site s. Define

$$
N^{s}(d) = \#\{k \in \mathbb{I} : |s-k| = d\}. \tag{C16}
$$

For a toroidal lattice this is the same for all sites s and hence $N^{s}(d) = N(d)$, however if the boundary conditions are not toroidal, then the number of neighbours at distance d will vary throughout the lattice. The general calculation of the total rate of change of a XY with general boundary conditions is therefore

$$
\frac{d}{dt}\mathbb{E}[\Delta P_{XY}] = \sum_{d=1}^{d_{\max}} \sum_{s\in\mathbb{I}:I(s)=1} \frac{\Delta P_{XY}(d)(E(s)|E) d_{\Theta}(s)}{N^{s}(d)} + \sum_{s\in\mathbb{I}:I(s)=0} \frac{\Delta P_{XY}(d)(E(s)|E) b_{\Theta}(s)}{N^{s}(d)}. \tag{C17}
$$

Appendix D: Data extraction

Original aerial photographic data of seagrass meadows from the Isles of Scilly, UK can be extracted from `http://www.channelcoast.org`. A full outline of the image processing can be found in[1]. The original aerial photographs from which the occupancy data was produced are in Fig. A3.

Appendix E: Metropolis Hastings algorithm derivation

We follow the derivation of Chib *et. al.*[2]. In order to sample parameters θ from a target distribution $\pi(\theta)$. The task is to construct an ergodic Markov chain with a stationary distribution $\pi(\theta)$. First, we define a proposal distribution $q(\theta'|\theta)$, that is the probability of proposing θ' given θ. In order for the reversibility condition to hold we require that

$$\pi(\theta)q(\theta'|\theta) = \pi(\theta')q(\theta|\theta') \tag{E1}$$

This, however would not generally be satisfied for a general form of q. We therefore define a probability of accepting the new value θ' given θ as $\alpha(\theta'|\theta)$ and therefore the probability of sampling θ' given θ is $\alpha(\theta'|\theta)q(\theta'|\theta)$. We therefore modify Eq. E1 with this new definition to produce

$$\pi(\theta)q(\theta'|\theta)\alpha(\theta'|\theta) = \pi(\theta')q(\theta|\theta')\alpha(\theta|\theta') \tag{E2}$$

Equivalently Eq. E2 may be written as

$$\frac{\alpha(\theta'|\theta)}{\alpha(\theta|\theta')} = \frac{\pi(\theta')q(\theta|\theta')}{\pi(\theta)q(\theta'|\theta)} \tag{E3}$$

Eq. E3 may be satisfied with the following choice of probability of acceptance

$$\alpha(\theta|\theta') = \min\left\{1, \frac{\pi(\theta')q(\theta|\theta')}{\pi(\theta)q(\theta'|\theta)}\right\} \tag{E4}$$

Due to the construction of the Markov chain, it is guaranteed to have the stationary distribution π[3].

References

[1] Jackson, E. *et al.* Isles of scilly seagrass mapping. Tech. Rep. 087, Natural England Commissioned Reports (2011).

[2] Chib, S. & Greenberg, E. Understanding the metropolis-hastings algorithm. *The american statistician* **49**, 327–335 (1995).

[3] Smith, A. F. & Roberts, G. O. Bayesian computation via the gibbs sampler and related markov chain monte carlo methods. *Journal of the Royal Statistical Society. Series B (Methodological)* 3–23 (1993).

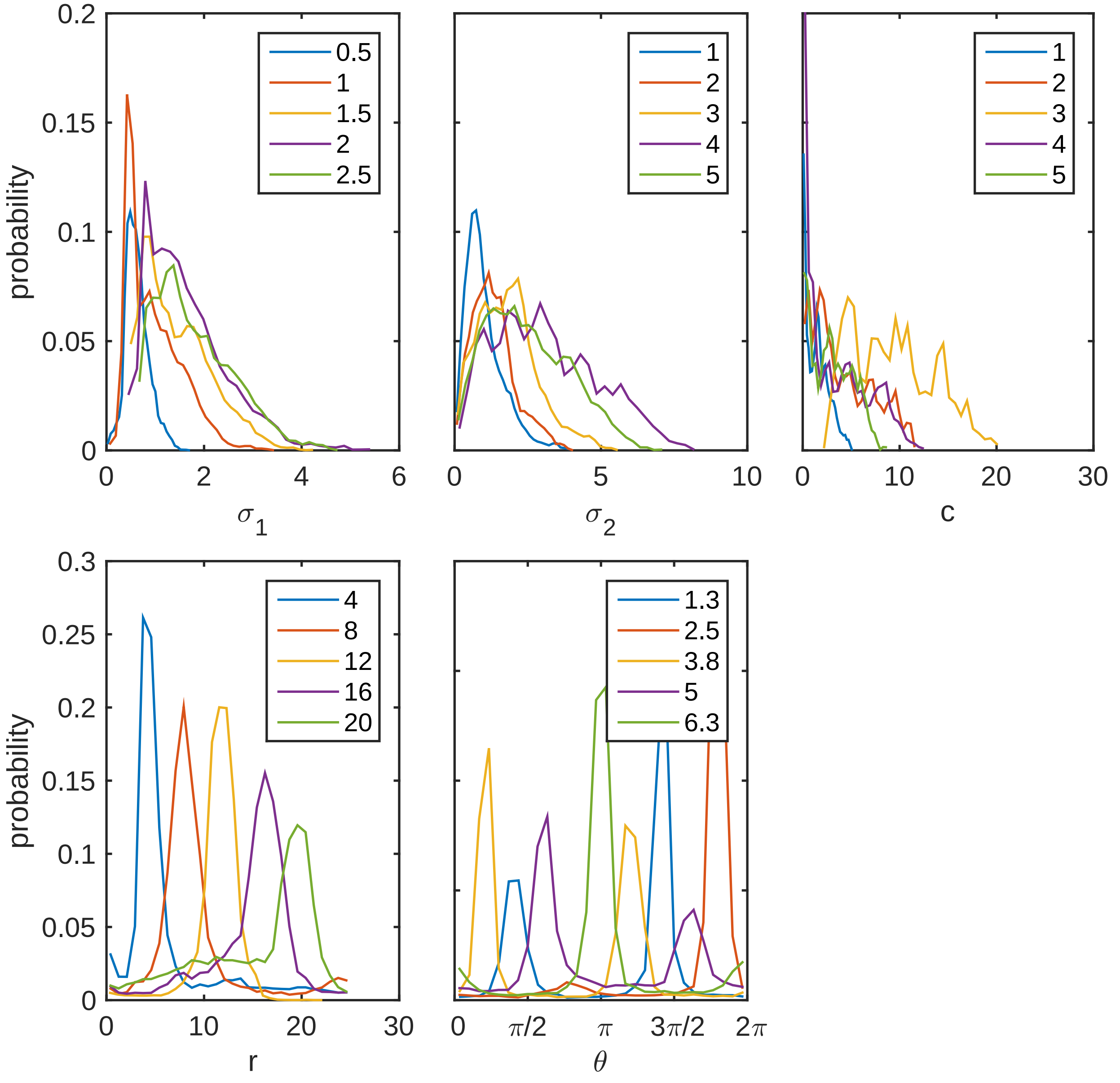

Figure A1: The marginal posterior for all parameters of the model where each parameter is varied in turn from the baseline example ($r = 10, \theta = \pi/2, \sigma_1 = 0.6, \sigma_2 = 2, c = 1$).

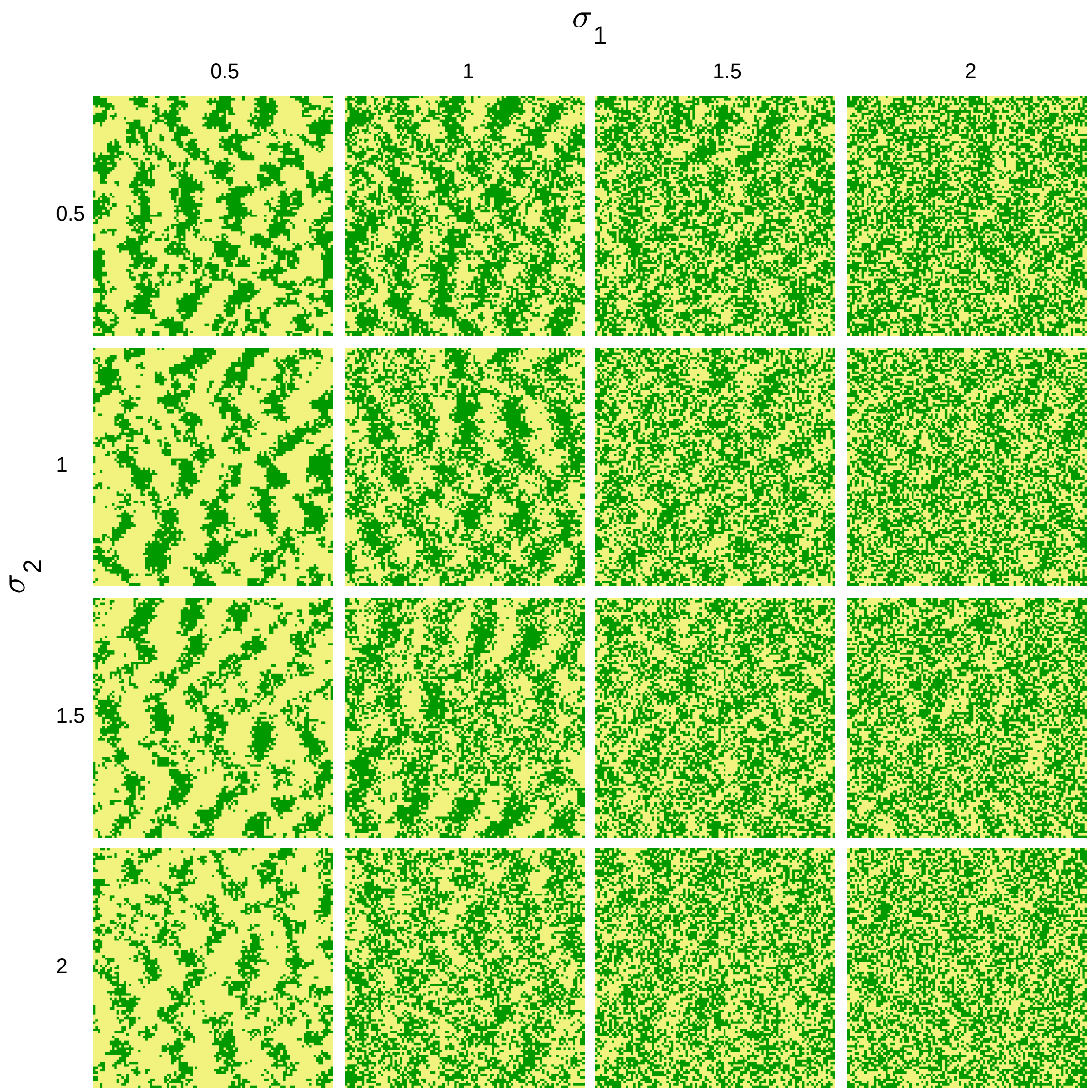

Figure A2: Snapshots at statistical equilibrium for increasing values of the reproduction and competition variance σ_1 and σ_2. When both are small, banding is able to form. However there is a destruction of the banded pattern and thus the correlation structure for larger σ_1 and σ_2. This makes it difficult to distinguish between values of the variances when they are greater than where banding can be sustained.

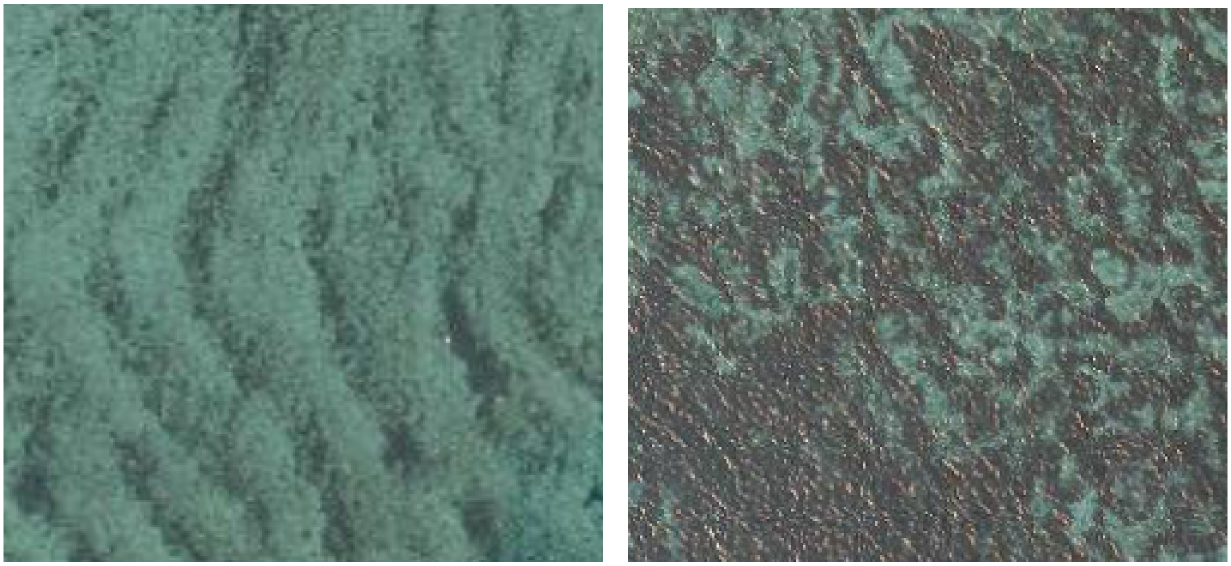

(a) (b)

Figure A3: Aerial photographs (100m$\times$100m) of two banding sites used as case-studies in the model fitting. False colouration was used to highlight the occupancy of seagrass.
